# Investigation of the Degree of Functionalization and Colloidal Stability of *Shell‐by‐Shell* Functionalized TiO_2_ Nanoparticles as a Function of Different Phosphonic Acid Chain Lengths

**DOI:** 10.1002/chem.202501008

**Published:** 2025-06-06

**Authors:** Lisa M. S. Stiegler, Vincent Wedler, İdil Büküşoğlu, Andreas Hirsch, Wolfgang Peukert, Johannes Walter

**Affiliations:** ^1^ Institute of Interfaces and Particle Technology (IPT) Friedrich‐Alexander‐Universität Erlangen‐Nürnberg (FAU) Cauerstraße 4 91058 Erlangen Germany; ^2^ Interdisciplinary Center for Functional Particle Systems (FPS) Friedrich‐Alexander‐Universität Erlangen‐Nürnberg (FAU) Haberstraße 9a 91058 Erlangen Germany; ^3^ Department of Chemistry and Pharmacy, Chair of Organic Chemistry II Friedrich‐Alexander‐Universität Erlangen‐Nürnberg (FAU) Nikolaus‐Fiebiger‐Straße 10 91058 Erlangen Germany

**Keywords:** AUC, colloidal stability, grafting density, Shell‐by‐Shell (SbS)‐functionalization, TGA

## Abstract

In this work, a series of *Shell‐by‐Shell* (*SbS*)‐functionalized colloidal systems consisting of 6 nm TiO_2_ anatase nanoparticles (NPs), one of the phosphonic acids (PAs) propylphosphonic acid (PAC_3_), hexylphosphonic acid (PAC_6_), dodecylphosphonic acid (PAC_12_), tetradecylphosphonic acid (PAC_14_), hexadecylphosphonic acid (PAC_16_), octadecylphosphonic acid (PAC_18_) and the amphiphile sodium dodecylbenzenesulfonate (SDBS) were prepared, resulting in TiO_2_‐PAC_X_@SDBS (X = 3, 6, 12, 14, 16, or 18) NPs dispersed in deionized water (DIW). During the whole functionalization process, the NPs were subjected to thermogravimetric analysis (TGA) to gain insights into their degree of functionalization and respective thermal stability. In addition, the colloidal stability of the NPs as a function of PA chain length was analyzed by analytical ultracentrifugation (AUC). By combining both TGA and AUC, it was found that the resulting hierarchical NP architectures form agglomerates, with the degree of agglomeration depending on the length of the PA used for the first‐shell functionalization. This allows conclusions to be drawn about the efficiency of the overlap between the PA and the SDBS carbon chains.

## Graphical abstract

The *Shell‐by‐Shell* structures of TiO_2_ nanoparticles, phosphonic acids (PAs) and sodium dodecylbenzenesulfonate (SDBS) in their solid and colloidal state were studied by thermogravimetric analysis and analytical ultracentrifugation. This combination showed that agglomerates were formed whose size depended on the PA chain length. This allowed conclusions to be drawn about the efficiency of the overlap between the PA and SDBS.

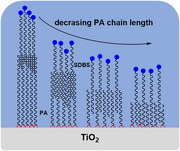

## 1 Introduction

Titanium dioxide nanoparticles (TiO_2_ NPs) are popular nanomaterials in research, development, and application due to their unique characteristics, which lie in their crystal, optical, and electrochemical properties. TiO_2_ exists in three major polymorphic forms, namely rutile, anatase, and brookite, which all have different crystal properties. The widespread use of TiO_2_ NPs in optical devices is due to their excellent mechanical resistance, their high transparency in the visible range and their chemical stability in aqueous media.^[^ ^1^ ^]^ TiO_2_ is a wide band gap n‐type semiconductor with indirect energy band gaps of 3.02, 3.2, or 2.96 eV for rutile, anatase, or brookite, respectively.^[^ ^2^ ^]^ Besides, anatase has a lower effective electron mass than rutile, which leads to a higher mobility of the charge carriers in anatase, a property that is very favorable for applications in optoelectronic devices.^[^ ^3^ ^]^ Great success has been achieved in the development of dye‐sensitized solar cells based on TiO_2_ anatase working as a n‐type semiconductor.^[^ ^4^ ^]^ Surface ligands are crucial for the application, synthesis, and processing of NPs.^[^ ^5^ ^]^ Since NPs are often handled as dispersions, surface ligands prevent them from Ostwald ripening, aging, and should have a positive influence on the colloidal stability.^[^ ^6^ ^]^ For the colloidal stability of NPs, it is crucial that the ligand is firmly bound to the NP surface so that no desorption occurs. In case of metal oxide NPs, phosphonic acids (PAs) are known be among the most stable anchor groups.^[^ ^7^ ^]^ For example, Lewis acidic TiO_2_ is a promising material for functionalization with PAs, as the corresponding bonds are among the strongest between metal oxides and ligands.^[^ ^7^, ^8^ ^]^ Recently, studies on the ligand exchange reactions of PAs onto TiO_2_ NP surfaces have been conducted.^[^ ^9^ ^]^ In terms of colloidal stability, the role of solution temperature, the PA chain length, and ligand tail structure as well as the PA surface coverage were analyzed.^[^ ^10^ ^]^ Applications, in which NPs are used as substrates for the construction of hierarchical architectures are highly interesting. This allows them to be functionalized through self‐assembly interactions with not only one type of ligand but several ligands. The beginnings in the fabrication of so‐called “*Layer‐by‐Layer* (*LbL*)” assemblies have been described by decher et al.^[^ ^11^ ^]^ In this approach, substrates have been modified either by acidification or with polyelectrolyte layers to create either positively or negatively charged surfaces.^[^ ^12^ ^]^ Subsequently, the charged substrates are alternately immersed in charged polymer solutions in order to build up multilayer arrays on the substrates through electrostatic interactions between oppositely charged building blocks.^[^ ^13^ ^]^ In view of the latter, the *LbL* technique represents a very simple, but efficient method for the sequential deposition of individual molecular layers on a planar solid support.^[^ ^14^ ^]^ In the meantime, numerous modifications on the above described *LbL* technique have been developed, e.g., for the construction of proof‐of‐concept solar cells^[^ ^12^, ^15^ ^]^ or the fabrication of photonic crystals.^[^ ^16^ ^]^ The processing to the *LbL* product is usually carried out using wet chemistry by immersing the modified substrate in charged polymer solutions. However, the substrates are dried after each layer applied so that the product itself is of solid state.^[^ ^17^ ^]^ Inspired by the *LbL* method is the so‐called “*Shell‐by‐Shell* (*SbS*)” method which is also carried out by wet chemistry, but the product exists in dispersed from, unlike the *LbL* product. Furthermore, the *SbS*‐functionalization is not based on electrostatic interactions as in the *LbL* method, but on two well‐defined functionalization steps. Within the first step, metal oxide NPs are functionalized with a hydrophobically terminated PA creating a self‐assembled monolayer (SAM) on top of the NP surface. During the second step, the first‐shell functionalized NPs are implemented with an amphiphile in deionized water (DIW), which forms a micellar arrangement around the NPs, driven by hydrophobic interactions.^[^ ^18^ ^]^

The second functionalization step within the *SbS*‐functionalization process is also known as “polarity umpolung” and describes the transformation of the dispersibility behavior from apolar solvents to water of initially hydrophobic coated NPs by the supramolecular noncovalent attachment of the amphiphile.^[^ ^19^ ^]^ The origins of the *SbS*‐functionalization technique date back to 1980, when shimoiizaka et al. used SDBS as a secondary adsorption layer to produce water‐based magnetic fluids. The further scientific process around the two‐step functionalization can also be found in the review by stiegler et al.^[^ ^18^ ^]^ Also of interest is the 2010 review by sperling and parak, in which they describe general strategies for the phase transfer of NPs from nonpolar to aqueous media by the addition of an extra layer of amphiphilic ligand molecules or amphiphilic polymers consisting of a hydrophobic side chain and a hydrophilic backbone.^[^ ^20^ ^]^ Furthermore, mulvaney, parak, and co‐workers presented different polymer encapsulation strategies for dispersing QDs in water, which are used as a universal tool for biolabeling experiments.^[^ ^21^ ^]^ A general discussion of electrostatic and steric stabilization of ultrasmall particles including quantum dots via stability maps can be found in the work by segets et al.^[^ ^22^ ^]^

The main advantage of this functionalization method is its generalizable route to switchable polarity reversal in various orthogonal solvents such as water, polar and apolar hydrocarbons, but also fluorocarbons.^[^ ^23^ ^]^ The adjustable dispersibility depends solely on the choice of PA and amphiphile as well as the solvent of choice.

So far, such *SbS*‐structured NPs were studied in terms of their dispersibility and surface wetting,^[^ ^23^ ^]^ optical and opto‐electronic properties,^[^ ^24^ ^]^ electron donor‐acceptor properties^[^ ^25^ ^]^ and their biomedical benefits.^[^ ^26^ ^]^

However, there are disadvantages to this method of functionalization. When functionalizing with an amphiphile, it is usually added in excess. In the next step, the particles are washed to remove excess amphiphile. Due to this, the NP dispersion is centrifuged and redispersed in DIW. The exact amount of amphiphile remaining on the NP surface can then only be determined inaccurately, also because there is a certain thermodynamic equilibrium between amphiphile attached to the surface and free amphiphile in solution. In addition, each additional washing step results in a loss of amphiphile as it is not covalently bound to the surface. Above a certain level of amphiphile loss, colloidal stability is also reduced due to reduced electro‐steric interactions. Another disadvantage is that the stability of these systems is highly dependent on the composition of PA, amphiphile, and solvent. The combination of these must be chosen carefully, otherwise the stability may be reduced. Once the solvophobic driving force of the amphiphile is inhibited in the solvent of choice, the amphiphile is released from the surface and the particles usually become unstable.

Here, we report on our approaches to study the functionalization degree including the associated thermal stability as well as the colloidal stability of *SbS*‐functionalized NPs as a function of the PA chain lengths and thus the potential chain overlap between PA and the amphiphile sodium dodecylbenzenesulfonate (SDBS), which determines the degree of agglomerates formed.

SDBS is used here instead of SDS, for example, because SDBS has already been established in previous work.^[^ ^23^ ^]^ It was originally used to track the absorption properties within the *SbS*‐structures due to the characteristic absorption band of the benzene structure. However, the stability of *SbS*‐functionalized systems is not expected to differ between SDS and SDBS, so the benzene structure should have no influence here.

## 2 Results and Discussion

### 2.1 Thermogravimetric Analysis

The starting point of our thermogravimetric analysis (TGA) studies was to find out what concentrations were required for the functionalization of our 6 nm anatase NPs (see Figure S4b) to achieve complete surface coverage. To establish comparability of all six PAs, we decided to functionalize all particle systems with the same concentration of PA. To determine the maximum monolayer grafting density, we chose a medium length PA, PAC_14_, as a prototypical system. The resulting concentration required to completely cover the surface was then applied to the other five systems.

Figure S2 shows the TGA measurements of TiO_2_‐PAC_14_ NPs functionalized with different concentrations of PAC_14_, where increasing weight loss was achieved with increasing concentration. The grafting densities were calculated using the following formula:^[^ ^27^ ^]^

(1) $$\sigma =\frac{wt}{100\%-wt}*\frac{N_{A}}{M_{W}*SSA}$$

including the weight loss (*wt*) in % for a given concentration used for functionalization, the Avogadro constant (*N*_A_), the molecular weight (*M*_W_) of the respective PAs, and the specific surface area (SSA). The SSA was determined by BET measurements, where the average value of (206 ± 5.67) m^2^/g was calculated from three measurements (see Table S4).

By plotting the observed grafting densities against the PAC_14_ concentrations, an asymptotic fit (*y* = *a*‐b*c^x^) could be applied, reflecting the Langmuir isotherm, as shown in Figure S3a. The asymptotic curve displays that the grafting density hardly changes from about 6.25 mM PAC_14_. Therefore, this concentration was used for all subsequent functionalization procedures. The reciprocals of the grafting densities and concentrations were also plotted using a linear fit, as shown in Figure S3b. The intercept of the y‐axis is the reciprocal of the grafting density, which corresponds to the maximum monolayer grafting density. This was calculated to be 4.9 molecules/nm^2^, which means that there are almost 5 PA molecules per square nanometer of TiO_2_ surface. This experimental result is in good agreement with literature values, where maximum monolayer grafting densities of PAC_16_ reached values of 3.7 molecules/nm^2^ for anatase TiO_2_ NPs,^[^ ^27^ ^]^ 5.7 molecules/nm^2^ for ferromagnetic Fe_3_O_4_ NPs,^[^ ^28^ ^]^ or 6.3 molecules/nm^2^ for Al_2_O_3_ NPs.^[^ ^29^ ^]^

**Figure 1:**
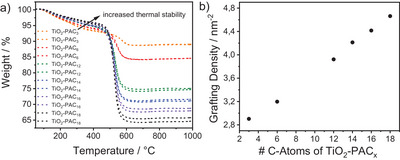
a) TGA measurements of TiO_2_‐PAC_X_, x = 3, 6, 12, 14, 16, 18 (double determination) and b) corresponding grafting densities calculated from the averaged weight losses.

**Scheme 1:**
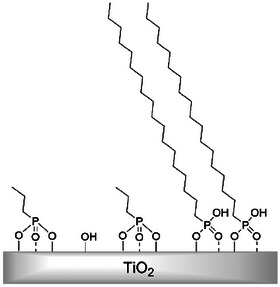
Overview of the surface packing density as a function of PA chain length.

By calculating the grafting densities from the average weight losses of both measurements, which were performed for reproducibility reasons for all six systems functionalized with the same PA concentration of 6.25 mM (see Figure 1a), an interesting trend was observed, as presented in Figure 1b. The grafting densities increased linearly with increasing PA chain length. This allows conclusions to be drawn about the surface packing density of the PAs on the TiO_2_ NPs. It appears that the PAs with shorter chains are bound to the substrate in a more multidentate manner than the PAs with longer chains. The latter appear to be less dentate. Analogous to our TGA results, okada et al. studied the functionalization degree of PAs of different chain length on TiO_2_ NPs by elemental analysis.^[^ ^10a^ ^]^ In agreement with our results, the elemental analysis also shows that the functionalization degree increases with increasing chain length, which in turn supports our hypothesis based on the decreasing denticity with increasing PA chain length. Scheme 1 presents a schematic illustration of the surface packing density as a function of the length of the PA chain. The concentration of 6.25 mM, that was experimentally determined for maximum monolayer surface coverage of PAC_14_, already represents a significant molar excess, as mathematically less PA is required to completely cover the surface. In other words, because the PA is already present in excess at 6.25 mM, higher grafting densities can be achieved than with PAC_14_. The monolayers formed by longer PA chains show higher surface coverage due to stronger van der Waals interactions between the chains, which increase with increasing chain length. It was also found that the thermal stability between the six systems increased with increasing PA chain length, which is particularly evident in Figure 1a in the diagonal increase of the weight loss curves between 200 °C and 450 °C. The trend of the improved thermal stability with increasing alkyl chain length has been reported in the literature and can also be explained by enhanced van der Waals interactions between neighboring alkyl chains.^[^ ^30^ ^]^

**Figure 2:**
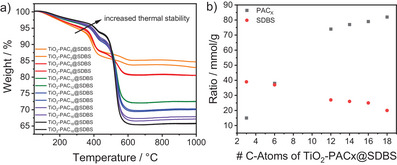
a) TGA measurements of TiO_2_‐PAC_X_@SDBS, x = 3, 6, 12, 14, 16, 18 (double determination) and b) corresponding ratios of PAC_X_ and SDBS calculated from the averaged weight losses.

**Scheme 2:**
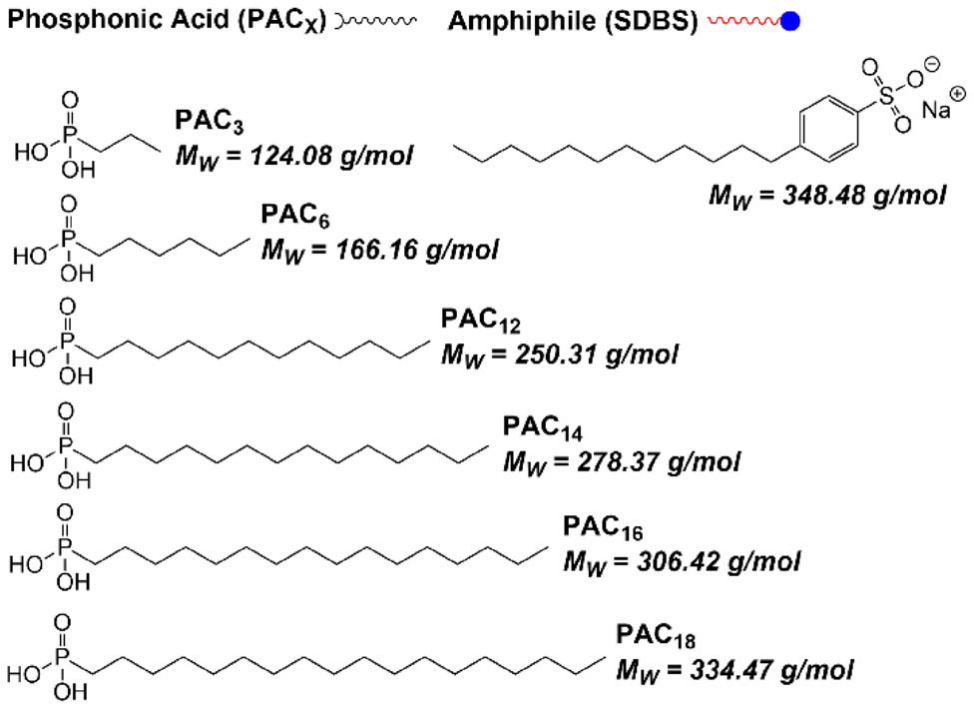
Overview of the used building blocks for the *SbS*‐functionalization.

In the next step, the TiO_2_ NPs functionalized with PAs of different chain lengths as first ligand shell building blocks and SDBS as second ligand shell building block were subjected to TGA. Figure 2a displays the TGA results of the six samples, each of which was measured twice to ensure the reproducibility of the results. For all samples, the first decrease observed in the weight loss curves can be attributed to the decomposition of the second ligand shell, while the subsequent, more pronounced decrease can be attributed to the decomposition of the first ligand shell. The second shell decomposed at lower temperatures than the first shell due to the noncovalent attachment of SDBS, which is mainly formed by hydrophobic interactions. In contrast, the covalent attachment of PA molecules to the NP surface results in a more stable monolayer, leading to the decomposition of this first ligand shell at higher temperatures. To gain an accurate understanding of the influence of both shells on the thermal decomposition, it was crucial to consider the molecular weight of the PAs and SDBS (see Scheme 2) together with the average values of the experimentally determined weight losses obtained from the TGA measurements. According to the given equations, the ratios of the ligand shells were determined for each *SbS*‐system and are presented in Table S7.

(2) $$X_{\mathrm{PA}C_{x}}=\frac{wt_{\mathrm{PA}C_{x}}}{{M_{W}}_{\mathrm{PA}C_{x}}}$$

(3) $$X_{\mathrm{SDBS}}=\frac{wt_{\mathrm{SDBS}}}{{M_{W}}_{\mathrm{SDBS}}}$$

**Scheme 3:**
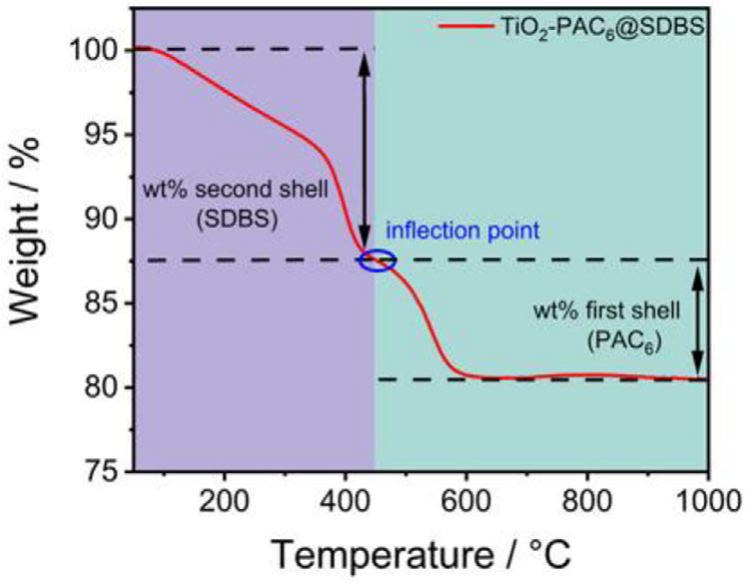
Graphical representation of the calculation of the ratio of PAC_6_ to SDBS using the weight loss curve of TiO_2_‐PAC_6_@SDBS as an example.

How the respective weight losses of PA and SDBS were obtained from the corresponding TGA curves of the *SbS*‐functionalized systems is shown graphically in Scheme 3 using TiO_2_‐PAC_6_@SDBS as an example.

Figure 2b depicts the plot of the calculated ratios of the first and second ligand shells as a function of the PA chain length. The increasing ratios of the first shell indicate a higher monolayer surface coverage with increasing PA chain length, which was already confirmed by the increasing grafting densities for first‐shell functionalized NPs (see Figure 1b) due to stronger van der Waals interactions between longer chains. In contrast, the second shell ratios show a significant decrease with increasing PA chain length. A possible explanation for this decrease is the formation of agglomerates during the *SbS*‐functionalization process, which progressively increased in size with increasing PA chain length, thus providing less surface area for the SDBS within the nanostructures.

The ligand packing density of a ligand on a solid is defined as the cross‐sectional area per chain divided by the available surface area per chain and is unaffected by NP core size or agglomerate size, but decreases with radial distance from the surface.^[^ ^31^ ^]^ However, as agglomerate size increases, the ligand deflection angle decreases and approaches values comparable to those of ligands functionalized on flat surfaces,^[^ ^32^ ^]^ which in turn hinders effective intercalation of SDBS. In addition, intercalation is sterically hindered in systems with longer PA chains because they have higher packing densities. Both reasons led to less SDBS being attached to the systems with longer PAs. As SDBS plays a key role in the electro‐steric stabilization of *SbS*‐functionalized particles in water, it is likely that systems with shorter PA chains will achieve better colloidal stability as they contain more SDBS.

To complete the characterization of the thermogravimetric properties of our six *SbS*‐functionalized samples, a further detailed look at Figure 2a should be made. Each curve shows two regimes of degradation, with the first degradation at lower temperatures corresponding to the second shell and the second degradation at higher temperatures corresponding to the first shell. It is clearly visible that the thermal stability of SDBS between 200 °C and 450 °C increases with increasing PA chain length. This is due to the fact that more thermal energy is required to overcome the stronger van der Waals interactions between longer PA chains and SDBS, which is necessary for the thermal decomposition of SDBS. Thus, it was observed that the thermal decomposition in the temperature range between 200 °C and 450 °C follows the same pattern for first‐shell and *SbS*‐functionalized NPs, both of which could be explained by van der Waals interactions.

### 2.2 Analysis of the Colloidal Stability

Once the thermogravimetric properties of such NP systems have been analyzed, the colloidally dispersed NPs were investigated. Here, we first analyzed the pristine NPs in DIW as received from the manufacturer and determined their zeta potential. It was found out to be (35.2 ± 3.0) mV (see Table S8) due to the addition of nitric acid as a stabilizing agent, which leads to an acidic pH. The electrostatic stabilization results in the NPs being present as individualized NPs, as demonstrated by analytical ultracentrifugation (AUC) measurements. The sedimentation coefficient distribution was obtained from the AUC experiments as displayed in Figure S4a and from this, the particle size distribution (see Figure S4b) was calculated by applying the stokes equation. The stokes equation, as shown below, applies only to spherical particles and includes the viscosity of the solvent (η), the sedimentation coefficient (s), the density of the NPs (ρ_NPs_) and the density of the solvent (ρ_Solvent_).

(4) $$x_{\mathrm{Stokes}}={\left(\frac{18\eta s}{\rho_{\mathrm{NPs}}-\;\rho_{\mathrm{Solvent}}}\right)}^{1/2}$$

This results in a size distribution with a maximum of 6.4 nm, which is in good agreement with the manufacturer´s size specification between 4 nm and 8 nm using DLS.

**Figure 3:**
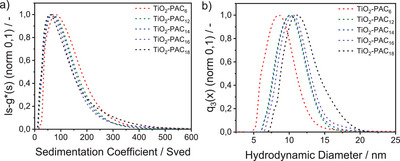
a) Sedimentation coefficient distributions and b) hydrodynamic diameters of TiO_2_‐PAC_X_, x = 3, 6, 12, 14, 16, 18 in toluene.

On the way to the *SbS*‐functionalized NPs, the first‐shell functionalized NPs were also investigated by AUC after the pristine ones. The resulting sedimentation coefficient distributions, displayed in Figure 3a, indicate that all the samples shown in the graph exhibited good colloidal stability and were well separated in toluene, with distributions ranging from 10 Sved to 400 Sved. However, a slight shift was observed between the sedimentation coefficient distributions, indicating that the NPs exhibited slower sedimentation rates with increasing PA chain length, thus shifting the sedimentation coefficient distributions to lower values. This behavior can be attributed to the different partial specific volumes (mean inverse total densities) of the NPs as well as to the hydrodynamic diameters, which were influenced by the different PA chain lengths, thus affecting their sedimentation coefficient distributions. The absence of TiO_2_‐PAC_3_ from the graph is due to the rapid sedimentation of the sample, which can be attributed to the extremely short PA chain, which was insufficient to provide adequate steric stabilization required to maintain colloidal stability. From the sedimentation coefficient distributions, the distributions of the core and hydrodynamic diameters were derived using an extended stokes equation, which considers the contribution of the shell to the overall particle density and size. The densities of the PAs were taken from the specifications of the manufacturers from whom the PAs were purchased, as follows: 1.132 g/cm^3^ for PAC_6_, 0.936 g/cm^3^ for PAC_12_, 0.998 g/cm^3^ for PAC_14_, 0.982 g/cm^3^ for PAC_16_, and 0.962 g/cm^3^ for PAC_18_. The values for the chain lengths were taken from literature, using values of 0.8 nm for PAC_6_, 1.5 nm for PAC_12_, 1.6 nm for PAC_14_, 1.8 nm for PAC_16_, and 2.1 nm for PAC_18_.^[^ ^33^ ^]^ These values were obtained from small‐angle x‐ray reflectometry (XRR) thickness measurements of n‐alkyl PA SAM/hafnium oxide (HfO_2_) hybrid dielectrics and thus from the solid state. Nevertheless, they could be transferred very well to our colloidal systems as presented in Figure 3b and Figure S5. Figure 3b depicts the resulting hydrodynamic size distributions of the TiO_2_‐PAC_X_ NPs, which show an increase in hydrodynamic diameter as a function of the chain length. To confirm the applied chain lengths, Figure S5 shows the particle size distributions of the NPs with the organic ligand shell subtracted, resulting in the same particle core size distributions for all five systems. This result confirms that all NPs have excellent colloidal stability and that the chain lengths obtained from the solid state can be transferred to the liquid phase.

**Figure 4:**
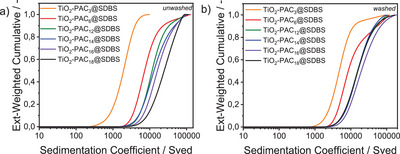
Logarithmically plotted extinction‐weighted cumulative sedimentation coefficient distributions of a) unwashed and b) washed TiO_2_‐PAC_X_@SDBS, x = 3, 6, 12, 14, 16, 18 in DIW.

Subsequently, the *SbS*‐functionalized NPs in DIW with SDBS as a second ligand shell, were subjected to AUC. The first series of samples was left unwashed, while the second series was washed after the addition of SDBS to remove excess SDBS. The aim of this approach was to compare the colloidal stability of the two variants across all six samples, primarily to assess the effect of excess SDBS in the NP dispersion on colloidal stability. The resulting sedimentation coefficient distributions of the unwashed and washed NPs in DIW are presented in Figures S6–S9 with their extinction‐weighted sedimentation coefficient distributions (measured at a wavelength of 320 nm) depicted in Figure 4a and b. The AUC experiments retrieved sedimentation coefficients much larger than those of the first‐shell functionalized NPs, indicating faster sedimentation due to NP agglomeration. The sedimentation coefficient distributions for both washed and unwashed samples show a similar trend, hence, shifting sedimentation coefficient distributions to higher values with increasing PA chain length, which is more pronounced in the unwashed samples. This can be explained with a loss of intercalated SDBS during the washing process, which leads to reduced functionalization and with this lowered colloidal stability of the washed samples. Among the unwashed samples (see again Figure 4a), the median s_50_ values increase with increasing PA chain length. Regarding the washed samples (see again Figure 4b), TiO_2_‐PAC_3_@SDBS NPs have the lowest s_50_ value, followed by TiO_2_‐PAC_6_@SDBS NPs, then very close together TiO_2_‐PAC_12_@SDBS, TiO_2_‐PAC_14_@SDBS and TiO_2_‐PAC_18_@SDBS, finally followed by TiO_2_‐PAC_16_@SDBS with the highest s_50_ value. The observed consistency of the results in the reproducibility measurements (see Figures S7 and S9) further supports this pattern.

As there were no single particles in the *SbS*‐functionalized NPs, in contrast to the first‐shell functionalized NPs, the sedimentation coefficient distributions were not converted into size distributions.

**Scheme 4:**
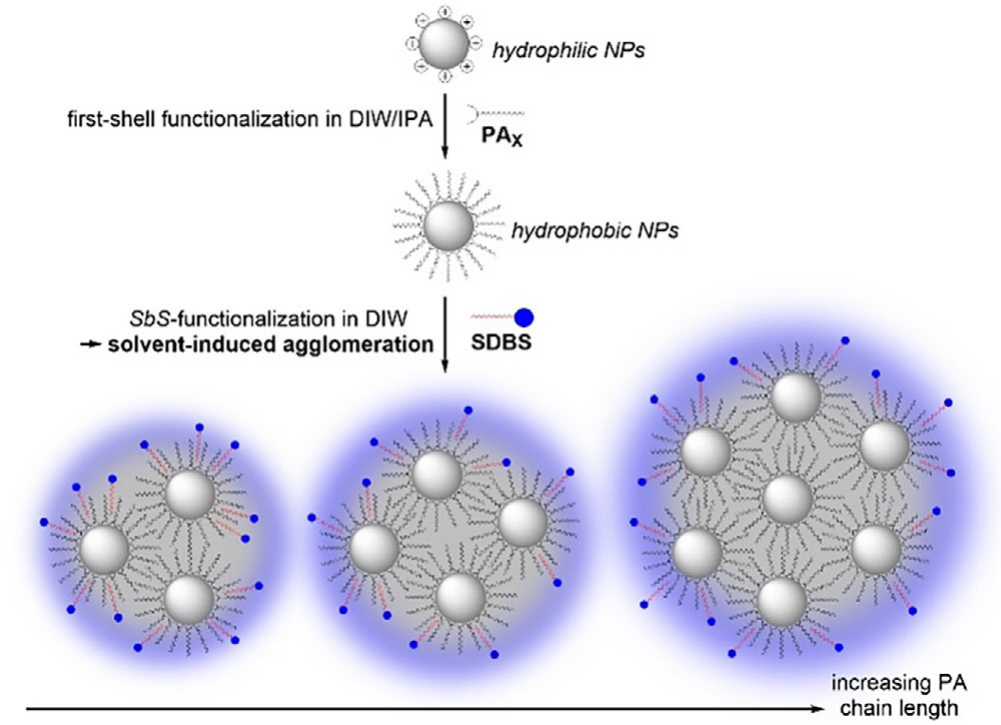
Overview of the conceivable formed agglomerate sizes during the *SbS*‐functionalization process in dependence on the PA chain length.

Overall, it can be deduced from the AUC results of the *SbS*‐functionalized NPs that the agglomerates become more pronounced with increasing PA chain length, as schematically shown in Scheme 4. The larger the agglomerates, the less SDBS is bound to the agglomerated particles, which is fully consistent with the results obtained from TGA, which showed a significant decrease in SDBS with increasing PA chain length (see Figure 2b).

The experimental results should now be considered in order to understand how agglomeration occurs. First, the hydrophobicity of the first‐shell functionalized NPs increases with increasing PA chain length until the NPs can even be classified as superhydrophobic above a certain chain length.^[^ ^34^ ^]^ In the next step, SDBS dissolved in water is added to the centrifuged first‐shell functionalized NPs. This step leads to solvent‐induced agglomeration due to the apolar‐polar interface, with increased agglomeration for particles with longer PA chain length. Because the surface is more densely packed with PA in systems with longer chains than in systems with shorter chains, the DIW is less able to interact with the hydrophilic TiO_2_ core of the particles.^[^ ^35^ ^]^ This in turn leads to more pronounced agglomeration. In addition, agglomeration provides less surface area for second shell functionalization, which in turn leads to an agglomeration‐induced decrease in SDBS with increasing degree of agglomeration.

Another effect that leads to less SDBS being bound to the systems with longer PA chains is the steadily increasing ligand packing density with increasing PA chain length, which prevents the SDBS from effective intercalation due to increasing steric hindrance. In this case, second‐shell functionalization is sterically facilitated for systems with shorter PA chain lengths. It has also been reported that NP stabilization is in principle also possible with pure amphiphiles and does not require an initial ligand shell.^[^ ^36^ ^]^

An effect that could be neglected, however, is the agglomeration induced by second‐shell functionalization, which occurs when SDBS does not lead to sufficient stabilization. In this case, agglomerates are formed after *SbS*‐functionalization, with the degree of agglomeration depending on the degree or efficiency of second shell functionalization. This effect can be neglected as there are numerous publications describing that SDBS or similarly structured amphiphiles led to a stabilization of such particles in water.^[^ ^37^ ^]^

From an energetic point of view, the second stage of *SbS*‐functionalization can be considered as follows. The addition of SDBS dissolved in water to the hydrophobic first‐shell functionalized NPs triggers the agglomeration due to the apolar‐polar interface. The agglomerates are in turn stabilized by SDBS through electro‐steric stabilizing effects. The driving force for SDBS to form such micelle‐like structures among the hydrophobic NP agglomerates in water is hydrophobic interactions. Since TGA measurements show that less SDBS remains on the NP agglomerates with longer PAs, the sum of hydrophobic interactions is lower than for the systems with shorter PAs. In addition to the hydrophobic interactions, van der Waals interactions between neighboring alkyl chains also determine the energetic state of the *SbS*‐structures. In this context, it was observed by TGA measurements that increased thermal stability was achieved for *SbS*‐systems based on longer PA chains, leading to the conclusion that van der Waals interactions are stronger between SDBS and longer‐chain PAs and weaker between SDBS and shorter‐chain PAs. The stronger van der Waals interactions in turn compensate to some extent for the lower hydrophobic interactions of the systems with longer PA chains and vice versa. Finally, entropy also plays an important role. Since in our case systems with longer PA chains tend to form larger agglomerates than systems with shorter PA chains, the entropy loss for the former system is greater because fewer possible microstates occur with increased agglomeration. Hydrophobic interactions, van der Waals interactions, and entropy loss all affect the energetic state of the systems. However, in order to compare the net free energy of the *SbS*‐systems composed of different PA chain lengths, we lack detailed calculations using molecular dynamics simulations.

## 3 Conclusion

Herein, we report our approaches to study the thermogravimetric properties and colloidal stability of *SbS*‐functionalized NPs as a function of PA chain length. TGA measurements were performed to gain insights into the degree of functionalization and the decomposition temperatures of the ligands used for the functionalization of TiO_2_ NPs. In the temperature range between 200 °C and 450 °C, the first‐shell functionalized NPs showed a higher thermal stability with increasing PA chain length and thus higher thermal sensitivity with decreasing PA chain length due to enhanced inter‐chain van der Waals interactions of longer chain PAs. In addition, the packing density of the monolayer increased with increasing PA chain length. For *SbS*‐functionalized NPs, it was shown that the decomposition of SDBS occurred prior to the decomposition of PA due to the noncovalent attachment of SDBS. SDBS also showed increased thermal stability between 200 °C and 450 °C with increasing PA chain length, again due to increased van der Waals interactions between longer chains. Interestingly, by TGA analysis, the amount of SDBS within the structure was observed to decrease with increasing PA chain length. This finding is supported by AUC measurements, which have shown that the *SbS*‐functionalized NPs form larger agglomerates as the PA chain length increases, resulting in less SDBS bound to the agglomerated particles. The decreasing amount of SDBS within the *SbS*‐structures can be explained by solvent‐induced agglomeration leading to increasing agglomerate sizes with increasing hydrophobicity of the particles with increasing PA chain length and thus less surface area for second shell functionalization. In addition, the increasing grafting densities with increasing PA chain length prevent effective overlap between PA and SDBS due to steric hindrance, which also leads to larger agglomerate sizes. Overall, this study provides an in‐depth understanding of the functionalization degree and agglomeration behavior of *SbS*‐functionalized NPs as a function of PA chain length. Such structures are now well enough understood to be used in a variety of applications and have already shown promise in biomedical applications.^[^ ^18^ ^]^ In the future, we hope that this functionalization method will be more widely used to produce customized NPs that can ultimately be used in therapeutic approaches, for example.

## 4 Experimental Section

### 4.1 Chemicals

All chemicals and solvents were purchased from the commercial suppliers Acros Organics (Fischer Scientific Loughborough, United Kingdom), Sigma‐Aldrich (Merck KGaA, Darmstadt, Germany), and VWR International (Rosny‐sous‐Bois, France) and were used, if not otherwise noted, without further purification. The solvents were used in HPLC grade. The TiO_2_ NPs (anatase) were obtained from PlasmaChem (Berlin, Germany) as a 20 wt% aqueous dispersion (product number: PL‐TiO‐20p‐, CAS‐No.: 1317–70–0).

### 4.2 Instrumentation

#### 4.2.1 Brunauer‐Emmett‐Teller (BET) Measurements

The BET measurements were performed on a Quadrasorb SI and a NOVAtouch from Quantachrome Instruments (now Anton Paar QuantaTec Inc., Boynton Beach, Florida, USA). Prior to BET measurements, the NPs were dried and degassed at a temperature of 200 °C and a pressure of 1.3 Pa for 2 hours with a heating ramp of 5 K/min. The measurements were based on nitrogen gas adsorption and SSAs were calculated by applying the multipoint BET method.

#### 4.2.2 TGA

TGA measurements were conducted using a TGA 8000 thermogravimetric analyzer from PerkinElmer (Waltham, USA) coupled to a Clarus 680 gas chromatograph from PerkinElmer and a Clarus SQ 8 C mass spectrometer. TGA was performed over a temperature range of 50 °C to 1000 °C with a N_2_ gas flow rate of 100 mL/min. The samples were initially maintained at 50 °C for 15 minutes to ensure the attainment of inert conditions, and subsequently heated from 50 °C to 1000 °C at a rate of 20 K/minute.

#### 4.2.3 Zeta‐Potential (ζ‐potential)

For ζ‐potential measurements, the Zetasizer Nano series ZEN3600 from Malvern Instruments (Herrenberg, Germany) was used. The device works with a 633 nm He‐Ne laser. The measurements were performed at 25 °C in PCS1115 glass cuvettes with an additional ZEN1002 dip cell.

#### 4.2.4 AUC

AUC experiments of the TiO_2_ NP dispersions were performed with a commercial analytical ultracentrifuge, type Optima AUC, from Beckman Coulter (Brea, California, USA). The initial 0.15 wt% NP dispersions in either toluene or DIW were further diluted to 0.0075 wt% to end up at an optical density of around 0.8 at 320 nm, measured with for a path length of 10 mm. Prior to each measurement, the NP dispersions were treated in an ultrasonic bath for 10 minutes. 400 µL of each sample was filled into a measuring cell from Nanolytics Instruments equipped with a two‐sector titanium centerpiece with a path length of 12 mm. The measurements of the pristine NPs in DIW and the first‐shell functionalized NPs in toluene were performed at a rotor speed of 10,000 rpm while the *SbS*‐functionalized NP dispersions in DIW were measured at a rotor speed of 3000 rpm. All measurements were conducted at a temperature of 20 °C. The sedimentation data were analyzed at a wavelength of 320 nm to have an optical density close to unity which guaranteed good signal within the Lambert‐Beer region. Data evaluation was carried out with the SEDFIT software (version 16.1c) using the least squares ls‐g*(s) method^[^ ^38^ ^]^ which provided the apparent sedimentation coefficient distributions. During sedimentation analysis, the radius and time‐invariant noise and the meniscus position were fitted. The sedimentation coefficient grid was logarithmically spaced (100 grid points) and the confidence level (F‐ratio) was set to 0.95.

For the conversion of the sedimentation coefficient distribution into the size distribution, the bulk density of TiO_2_ (3.78 g/cm^3^) was used as well as the respective densities and viscosities of DIW (0.99 820 g/cm^3^, 1.0016 mPa s) and toluene (0.86 690 g/cm^3^, 0.6000 mPa s) at 20 °C.

### 4.3 Nanoparticle Functionalization

#### 4.3.1 First‐Shell Functionalization

7.5 mL of a 0.25 wt% TiO_2_ NP dispersion in DIW was mixed with a solution of the corresponding PA (PAC_3_, PAC_6_, PAC_12_, PAC_14_, PAC_16_, PAC_18_) in isopropanol (IPA), ending up in a concentration of 6.25 mM PA in dispersion. The thus created 0.15 wt% NP dispersions were sonicated for 30 minutes, followed by three washing steps. Washing included centrifugation (11,000 rpm/15 minutes) of the dispersions with discarding the supernatant and redispersing the NP sediment in pure IPA in the ultrasonic bath. After the third washing step, the sedimented NPs were redispersed in 11.5 mL toluene, ending up in 0.15 wt% NP dispersions.

#### 4.3.2 SbS‐Functionalization

After the third washing step sedimented first‐shell functionalized NPs were redispersed in 10 mL of a 1 mM SDBS solution in DIW, followed by one washing step including centrifugation (11,000 rpm/15 minutes), discarding the supernatant and redispersing the NP sediment in 10 mL DIW, ending up in 0.15 wt% NP dispersions.

### 4.4 Sample Preparation

#### 4.4.1 Sample Preparation for TGA measurements

For TGA measurements, 0.15 wt% washed TiO_2_‐PAC_X_ and unwashed TiO_2_‐PAC_X_@SDBS NP dispersions were centrifuged (11,000 rpm/15 minutes), the supernatant discarded, and the NP sediment dried in a drying oven at 40 °C for 2 days.

#### 4.4.2 Sample Preparation for AUC Measurements

For AUC measurements, 0.15 wt% pristine, TiO_2_‐PAC_X_ and TiO_2_‐PAC_X_@SDBS NP dispersions (X = 3, 6, 12, 14, 16, or 18) were diluted to 0.0075 wt% dispersions.

## Conflict of Interests

The authors declare no conflict of interest.

## Supporting information



Supporting Information

## Data Availability

The data supporting the results of this study are available in the supplementary information to this article. All raw data used in this publication are available in Zenodo at https://doi.org/10.5281/zenodo.14644719, reference number 14 644 719.
