# Supplementary material for: Investigation of the Degree of Functionalization and Colloidal Stability of Shell‐by‐Shell Functionalized TiO2 Nanoparticles as a Function of Different Phosphonic Acid Chain Lengths
Source: Chemistry. 2025 Jun 6;31(37):e202501008. doi: 10.1002/chem.202501008 (PMC12223353; doi:10.1002/chem.202501008)
Supplement: Supplementary file 1 — Supporting Information [file CHEM-31-e202501008-s001.docx]

Supporting Information

**Investigation of the Degree of Functionalization and Colloidal Stability of *Shell-by-Shell* Functionalized TiO_2_ Nanoparticles as a Function of Different Phosphonic Acid Chain Lengths**

Lisa M. S. Stiegler^[a],[b]^, Vincent Wedler^[c]^, İdil Büküşoğlu^[a],[b]^, Andreas Hirsch^[c]^, Wolfgang Peukert^[a],[b]^ and Johannes Walter^[a],[b]*^

[a] Institute of Interfaces and Particle Technology (IPT), Friedrich-Alexander-Universität Erlangen-Nürnberg (FAU), Cauerstraße 4, 91058 Erlangen, Germany, e-mail: johannes.walter@fau.de

[b] Interdisciplinary Center for Functional Particle Systems (FPS), Friedrich-Alexander-Universität Erlangen-Nürnberg (FAU), Haberstraße 9a, 91058 Erlangen, Germany

[c] Department of Chemistry and Pharmacy, Chair of Organic Chemistry II, Friedrich-Alexander-Universität Erlangen-Nürnberg (FAU), Nikolaus-Fiebiger-Straße 10, 91058 Erlangen, Germany

**BET results**

***Table S1.*** *1^st^ BET measurement.*

| Surface Area Data |  |
| --- | --- |
| MultiPoint BET | 2.135e+02 m²/g |
| BJH method cumulative desorption surface area | 7.083e+00 m²/g |
| DH method cumulative desorption surface area | 7.224e+00 m²/g |
| t-method external surface area | 1.530e+01 m²/g |
| t-method micropore surface area | 1.982e+02 m²/g |
| DFT cumulative surface area | 1.610e+02 m²/g |
|  |  |
| Pore Volume Data |  |
| Total pore volume for pores with Radius  less than 1366.39 Å at P/Po = 0.992941 | 1.158e-01 cc/g |
| BJH method cumulative desorption pore volume | 8.627e-03 cc/g |
| DH method cumulative desorption pore volume | 8.447e-03 cc/g |
| t-method micropore volume | 9.518e-02 cc/g |
| HK method micropore volume | 9.450e-02 cc/g |
| SF method micropore volume | 8.467e-02 cc/g |
| DFT method cumulative pore volume | 1.032e-01 cc/g |
|  |  |
| Pore Size Data |  |
| Average pore Radius | 1.084e+01 Å |
| BJH method desorption pore Radius (Mode Dv(r)) | 1.572e+01 Å |
| DH method desorption pore Radius (Mode Dv(r)) | 1.572e+01 Å |
| HK method pore Radius (Mode) | 1.838e+00 Å |
| SF method pore Radius (Mode) | 1.754e+00 Å |
| DFT pore Radius (Mode) | 8.440e+00 Å |

***Table S2.*** *2^nd^ BET measurement.*

| Surface Area Data |  |
| --- | --- |
| MultiPoint BET | 2.020e+02 m²/g |
| BJH method cumulative desorption surface area | 6.861e+00 m²/g |
| DH method cumulative desorption surface area | 6.985e+00 m²/g |
| t-method external surface area | 1.409e+01 m²/g |
| t-method micropore surface area | 1.879e+02 m²/g |
| DFT cumulative surface area | 1.471e+02 m²/g |
|  |  |
| Pore Volume Data |  |
| Total pore volume for pores with Radius  less than 1459.13 Å at P/Po = 0.993394 | 1.062e-01 cc/g |
| BJH method cumulative desorption pore volume | 7.821e-03 cc/g |
| DH method cumulative desorption pore volume | 7.658e-03 cc/g |
| t-method micropore volume | 8.831e-02 cc/g |
| HK method micropore volume | 8.804e-02 cc/g |
| SF method micropore volume | 6.536e-02 cc/g |
| DFT method cumulative pore volume | 9.546e-02 cc/g |
|  |  |
| Pore Size Data |  |
| Average pore Radius | 1.052e+01 Å |
| BJH method desorption pore Radius (Mode Dv(r)) | 1.582e+01 Å |
| DH method desorption pore Radius (Mode Dv(r)) | 1.582e+01 Å |
| HK method pore Radius (Mode) | 1.838e+00 Å |
| SF method pore Radius (Mode) | 2.261e+00 Å |
| DFT pore Radius (Mode) | 8.068e+00 Å |

***Table S3.*** *3^rd^ BET measurement.*

| Surface Area Data |  |
| --- | --- |
| MultiPoint BET | 2.020e+02 m²/g |
| BJH method cumulative desorption surface area | 8.267e+00 m²/g |
| DH method cumulative desorption surface area | 8.417e+00 m²/g |
| t-method external surface area | 1.576e+01 m²/g |
| t-method micropore surface area | 1.862e+02 m²/g |
| DFT cumulative surface area | 1.419e+02 m²/g |
|  |  |
| Pore Volume Data |  |
| Total pore volume for pores with Radius  less than 2237.65 Å at P/Po = 0.995707 | 1.080e-01 cc/g |
| BJH method cumulative desorption pore volume | 9.441e-03 cc/g |
| DH method cumulative desorption pore volume | 9.242e-03 cc/g |
| t-method micropore volume | 8.755e-02 cc/g |
| HK method micropore volume | 8.661e-02 cc/g |
| SF method micropore volume | 6.468e-02 cc/g |
| DFT method cumulative pore volume | 9.705e-02 cc/g |
|  |  |
| Pore Size Data |  |
| Average pore Radius | 1.069e+01 Å |
| BJH method desorption pore Radius (Mode Dv(r)) | 1.574e+01 Å |
| DH method desorption pore Radius (Mode Dv(r)) | 1.574e+01 Å |
| HK method pore Radius (Mode) | 1.838e+00 Å |
| SF method pore Radius (Mode) | 2.261e+00 Å |
| DFT pore Radius (Mode) | 8.440e+00 Å |

- BET Summary

***Table S4.*** *Determined mean value of the specific surface area from BET measurements.*

| Measurement | Specific Surface Area | Unit |
| --- | --- | --- |
| 1) | 2.14E+02 | m²/g |
| 2) | 2.02E+02 | m²/g |
| 3) | 2.02E+02 | m²/g |
| Mean Value | 2.06E+02 | m²/g |
| SD | ± 5.67 | m²/g |

**TGA results**

***Figure S1.*** *TGA measurements of pristine TiO_2_, PAC_X_ (x = 3, 6, 12, 14, 16, 18) and SDBS.*

***Figure S2.*** *TGA measurements of TiO_2_-PAC_14_ functionalized with different concentrations of PAC_14_.*

***Table S5.*** *Concentrations, reciprocal values of concentrations, weight losses, calculated grafting densities and reciprocal values of grafting densities for TiO_2_-PAC_14_ functionalized with different concentrations of PAC_14_.*

| Concentration / mM | 1/Concentration / mM^-1^ | Weight Loss / % | Grafting Density / nm^-2^ | 1/Grafting Density / nm^2^ |
| --- | --- | --- | --- | --- |
| 0.50 | 2.00 | 14.79 | 1.82 | 0.55 |
| 0.75 | 1.33 | 17.03 | 2.15 | 0.46 |
| 1.00 | 1.00 | 19.40 | 2.53 | 0.40 |
| 1.25 | 0.80 | 20.32 | 2.67 | 0.37 |
| 1.50 | 0.67 | 22.88 | 3.12 | 0.32 |
| 1.75 | 0.57 | 25.15 | 3.50 | 0.29 |
| 2.00 | 0.50 | 25.92 | 3.67 | 0.27 |
| 2.25 | 0.44 | 26.04 | 3.67 | 0.27 |
| 2.50 | 0.40 | 28.23 | 4.12 | 0.24 |
| 3.75 | 0.27 | 27.02 | 3.86 | 0.26 |
| 5.00 | 0.20 | 28.77 | 4.23 | 0.24 |
| 6.25 | 0.16 | 30.05 | 4.48 | 0.22 |
| 7.50 | 0.13 | 28.65 | 4.21 | 0.24 |
| 8.75 | 0.11 | 29.85 | 4.46 | 0.22 |
| 10.00 | 0.10 | 29.71 | 4.44 | 0.23 |
| 15.00 | 0.07 | 29.60 | 4.42 | 0.23 |
| 20.00 | 0.05 | 30.64 | 4.63 | 0.22 |

***Figure S3.*** *a) Grafting densities for TiO_2_-PAC_14_ versus ligand concentration, with a fit of the Langmuir isotherm (asymptotic fit) for monolayer grafting of PAC_14_. b) Reciprocal values of grafting densities versus reciprocal values of ligand concentration, linearly fitted.*

***Table S6.*** *Average weight losses from two measurements and corresponding calculated grafting densities for TiO_2_-PAC_X_, x = 3, 6, 12, 14, 16, 18.*

|  | Weight Loss / % | Grafting Density / nm^-2^ |
| --- | --- | --- |
| TiO_2_-PAC_3_ | 10.97 | 2.90 |
| TiO_2_-PAC_6_ | 15.38 | 3.20 |
| TiO_2_-PAC_12_ | 25.14 | 3.92 |
| TiO_2_-PAC_14_ | 28.63 | 4.21 |
| TiO_2_-PAC_16_ | 31.64 | 4.42 |
| TiO_2_-PAC_18_ | 34.79 | 4.66 |

***Table S7.*** *Average weight losses from two measurements and corresponding calculated ratios of the first and second ligand shells for TiO_2_-PAC_X_@SDBS, x = 3, 6, 12, 14, 16, 18.*

|  | Weight PAC_x_ / % | Weight SDBS / % | Ratio (PAC_x_) / mmol/g | Ratio (SDBS) / mmol/g |
| --- | --- | --- | --- | --- |
| TiO_2_-PAC_3_@SDBS | 84.44 | 86.28 | 15 | 39 |
| TiO_2_-PAC_6_@SDBS | 80.65 | 86.88 | 38 | 37 |
| TiO_2_-PAC_12_@SDBS | 72.10 | 90.71 | 74 | 27 |
| TiO_2_-PAC_14_@SDBS | 69.60 | 91.03 | 77 | 26 |
| TiO_2_-PAC_16_@SDBS | 66.94 | 91.22 | 79 | 25 |
| TiO_2_-PAC_18_@SDBS | 65.41 | 93.05 | 82 | 20 |

**Zeta potential results**

***Table S8.*** *Zeta potential values of bare TiO_2_ NPs dispersed in DIW.*

|  | Zeta Potential / mV |
| --- | --- |
| 1^st^ measurement | 32.8 |
| 2^nd^ measurement | 34.1 |
| 3^rd^ measurement | 38.6 |
| Mean value | 35.2 |
| Standard deviation | 3.0 |

**AUC results**

***Figure S4.*** *a) Sedimentation coefficient distribution and b) particle size distribution of pristine TiO_2_ NPs in DIW.*

***Figure S5.*** *Particle size distribution of TiO_2_-PAC_X_ in toluene, x = 6, 12, 14, 16, 18.*

***Figure S6.*** *Sedimentation coefficient distributions of unwashed TiO_2_-PAC_X_@SDBS in DIW, x = 6, 12, 14, 16, 18.*

***Figure S7.*** *a**) Sedimentation coefficient distributions and b) logarithmically plotted extinction-weighted cumulative sedimentation coefficient distributions of unwashed TiO_2_-PAC_X_@SDBS in DIW, x = 6, 12, 14, 16, 18. Note: This is a reproducibility measurement to the experiment related to Figure S6.*

***Figure S8.*** *Sedimentation coefficient distributions of washed TiO_2_-PAC_X_@SDBS in DIW, x = 6, 12, 14, 16, 18.*

***Figure S9.*** *a) Sedimentation coefficient distributions and b) logarithmically plotted extinction-weighted cumulative sedimentation coefficient distributions of washed TiO_2_-PAC_X_@SDBS in DIW, x = 6, 12, 14, 16, 18. Note: This is a reproducibility measurement to the experiment related to Figure S8.*
